# Extracellular Vesicles Released From the Skin Commensal Yeast *Malassezia sympodialis* Activate Human Primary Keratinocytes

**DOI:** 10.3389/fcimb.2020.00006

**Published:** 2020-01-24

**Authors:** Helen Vallhov, Catharina Johansson, Rosanne E. Veerman, Annika Scheynius

**Affiliations:** ^1^Department of Clinical Science and Education, Karolinska Institutet, and Sachs' Children and Youth Hospital, Södersjukhuset, Stockholm, Sweden; ^2^Immunology and Allergy Unit, Department of Medicine Solna, Karolinska Institutet, and Karolinska University Hospital, Stockholm, Sweden; ^3^Science for Life Laboratory, Karolinska Institutet, Stockholm, Sweden

**Keywords:** extracellular vesicles, fungi, ICAM-1, keratinocytes, MalaEx, *Malassezia*

## Abstract

Extracellular vesicles (EVs) released from fungi have been shown to participate in inter-organismal communication and in cross-kingdom modulation of host defense. *Malassezia* species are the dominant commensal fungal members of the human skin microbiota. We have previously found that *Malassezia sympodialis* releases EVs. These EVs, designated MalaEx, carry *M. sympodialis* allergens and induce a different inflammatory cytokine response in peripheral blood mononuclear cells (PBMC) from patients with atopic dermatitis compared to healthy controls. In this study, we explored the host-microbe interaction between MalaEx and human keratinocytes with the hypothesis that MalaEx might be able to activate human keratinocytes to express the intercellular adhesion molecule-1 (ICAM-1, CD54). MalaEx were prepared from *M. sympodialis* (ATCC 42132) culture supernatants by a combination of centrifugation, filtration and serial ultracentrifugation. The MalaEx showed a size range of 70–580 nm with a mean of 154 nm using nanoparticle tracking analysis. MalaEx were found to induce a significant up-regulation of ICAM-1 expression on primary human keratinocytes isolated from human *ex vivo* skin (*p* = 0.026, *n* = 3), compared to the unstimulated keratinocytes. ICAM-1 is a counter ligand for the leukocyte integrins lymphocyte function-associated antigen-1 (LFA-1) and macrophage-1 antigen (Mac-1), of which induced expression on epithelial cells leads to the attraction of immune competent cells. Thus, the capacity of MalaEx to activate keratinocytes with an enhanced ICAM-1 expression indicates an important step in the cutaneous defense against *M. sympodialis*. How this modulation of host cells by a fungus is balanced between the commensal, pathogenic, or beneficial states on the skin in the interplay with the host needs to be further elucidated.

## Introduction

Extracellular vesicles (EVs) are released not only from different mammalian cell-types but also from microorganisms, parasites, and plants (Bielska et al., 2019). EVs are heterogenous in size, 20 up to 1,000 nm in diameter, and their function differs depending on their cell of origin, different routes involved in biogenesis, release and their cargo (Soares et al., 2017). They are mainly classified into two major groups based on size; exosomes of endosomal origin with a diameter up to 150 nm, and microvesicles bigger than 150 nm generated through outward budding of the plasma membrane (Thery et al., 2018). EVs from microorganisms, such as fungi, are usually around 100–1,000 nm, and have been associated with cytotoxicity, the invasion of host cells, and the transfer of virulence factors (Brown et al., 2015). Fungal EVs have been shown to participate in inter-organismal communication and in cross-kingdom modulation of host cells (Bielska and May, 2019) with their capacity to deliver functional (m)RNAs and micro (mi)RNA-like RNAs to recipient cells (Peres da Silva et al., 2015). Notably, EVs from the host have on their part been found to deliver host sRNA into fungal cells and induce cross-kingdom RNA interference (RNAi) to silence fungal virulence genes (Cai et al., 2018).

*Malassezia* species are the dominant fungal members of the human skin microbiota (Findley et al., 2013) and detectable already on the skin in 100% of newborns (Nagata et al., 2012). The genus *Malassezia* comprises a heterogenous group of increasing number of identified species, and novel culture independent methods suggest a wider-spread than previously described (Theelen et al., 2018). Although, a commensal skin colonizing yeast, *Malassezia* are also associated with a number of skin disorders including pityriasis versicolor, folliculitis, seborrheic dermatitis, dandruff, and atopic dermatitis (AD) (Saunders et al., 2012). Recently, it was found that M*alassezia restricta* is associated with the colonic mucosa in a subset of patients with Chrohn's disease who have a disease-linked polymorphism in *CARD9* (Limon et al., 2019). This observation is interesting in a host-microbe perspective indicating that host genetic factors may increase colonization with *Malassezia* and the inflammatory response.

For many years, we have utilized *Malassezia sympodialis*, the fungi most frequently colonizing the skin of both AD patients and healthy individuals in our part of the globe (Sandstrom Falk et al., 2005), as a model to investigate host-microbe interactions. We have discovered that *M. sympodialis* releases EVs, which we designated MalaEx (Gehrmann et al., 2011). These EVs are compared to their parental *M. sympodialis* whole yeast cells enriched in 110 proteins, among those two of the *M. sympodialis* allergens, Mala s 1 and Mala s 7 (Johansson et al., 2018), and they can induce inflammatory cytokine responses with a significantly higher IL-4 production in peripheral blood mononuclear cells (PBMC) from patients with AD sensitized to *Malassezia* compared to healthy controls (Gehrmann et al., 2011). Thus, MalaEx seem to play a contributing role in eliciting and maintaining eczema in patients with AD. Furthermore, we have in MalaEx detected several small RNAs with well-defined start and stop positions in a length range of 16–22 nucleotides (Rayner et al., 2017), suggesting their capacity to deliver either autocrine or paracrine signaling for *M. sympodialis* in the interplay with the host and the environment.

The first major cell population *Malassezia* interacts with on the skin is epidermal keratinocytes. Besides forming an effective mechanical barrier to the outer environment keratinocytes are also active components of the immunoregulatory network in the skin (Di Meglio et al., 2011). Keratinocytes produce and express several mediators, in response to outer signals and transmit those to immune cells in the skin thereby regulating skin immunity and inflammation (Pasparakis et al., 2014). Whole yeast cells from different *Malassezia* species have been found to induce release of a variable profile of inflammatory mediators by keratinocytes (Watanabe et al., 2001; Ishibashi et al., 2006; Donnarumma et al., 2014). Previously, we discovered an active binding and internalization of MalaEx into human keratinocytes, where MalaEx were mainly found in close proximity of the keratinocyte nuclei, suggesting a central communication with the host cell (Johansson et al., 2018). In this study, we explored the host-microbe interaction between MalaEx and human keratinocytes with the hypothesis that MalaEx might be able to activate human keratinocytes to express the intercellular adhesion molecule-1 (ICAM-1, CD54).

## Materials and Methods

### *Malassezia sympodialis* Culture Conditions

*M. sympodialis* (ATCC 42132) was cultured on Dixon agar plates (Gueho et al., 1996) modified to contain 1% (vol/vol) Tween 60, 1% (wt/vol) agar, and no oleic acid (mDixon) at 32°C. After 2 or 4 days, the yeast cells were harvested, re-suspended and washed in PBS. Counting in a Bürker chamber using trypan blue exclusion showed a viability above 95%. From the 4 days cultures 6 × 10^7^ live yeast cells/ml were added to RPMI 1640 medium supplemented with penicillin 100 units/ml, streptomycin 100 μg/ml, 2 mM L-glutamine, and 10% heat inactivated fetal calf serum (all from Gibco BRL, Life Technologies Ltd, Paisley, UK) and incubated for 48 h in 6% CO_2_ at 37°C, as previously described (Gehrmann et al., 2011). Prior usage, fetal calf serum had been ultra centrifuged overnight at 100 000 × g followed by filtration through a 0.22 μm filter (Nordic Biolabs, Täby, Sweden) to remove possible EV contaminants. Each culture batch consisted of 320 ml distributed over four 175 cm^2^ flasks (Falcon, Corning Inc., Tewksbury, MA, USA). At each culture step blood and Sabouraud agar plates were inoculated in parallel to exclude bacterial and *Candida* contaminations, respectively.

### MalaEx Preparation

MalaEx were prepared from the 48 h *M. sympodialis* culture supernatants by using a combination of centrifugation, filtration, and serial ultracentrifugation. The culture supernatants were spun at 1 200 × g for 5 min followed by 3 000 × g for 30 min, and thereafter filtered through a 0.22 μm filter (Nordic Biolabs) and frozen to −80°C until further preparation. After thawing at RT, supernatants were centrifuged at 10 000 × g for 30 min. Thereafter, MalaEx were pelleted from the supernatants at 100 000 × g for 90 min, re-suspended in PBS and pelleted again at 100 000 × g for 90 min. The resulting pellet was carefully re-suspended in 100 μl PBS and stored frozen at −80°C. To avoid batch variations and to obtain enough MalaEx for the stimulation experiments (see below) three MalaEx preparations were pooled. The protein content was measured using a detergent compatible (DC) protein assay according to the manufacturer's instructions (BioRad, Hercules, CA, USA).

### Nanoparticle-Tracking Analysis (NTA)

The particle size and concentration of the pooled MalaEx preparation was measured using a LM10 platform with sCMOS camera from NanoSight Ltd, Amesbury, UK. The system is equipped with a 405 nm laser running nanoparticle tracking analysis (NTA) 2.3 analytical software package. The samples were diluted 3,000 × in 30 kDa filtered PBS and analyzed with camera level 14 and detection threshold 7. Four consecutive videos were recorded in RT while injecting the sample with a syringe pump (speed 50). The result is expressed as the mean particle size ± SD and the particle concentration of the four separate NTA runs of the pooled MalaEx sample.

### Preparation of Human Primary Keratinocytes

Epidermal keratinocytes were isolated according to the manufacturer's instructions (Gibco Invitrogen Corporation, Paisley, UK) from human abdomen *ex vivo* skin received from a local plastic surgery clinic. In short, thin skin tissues were prepared by using a dermatome, which thereafter were incubated in a Dispase solution (25 caseinolytic units/ml; Gibco Invitrogen Corporation) for 18 h at 4°C. Epidermis was separated from dermis and placed into 0.05% Trypsin-EDTA (Gibco Invitrogen Corporation) for 15 min at 37°C for cell dissociation. After addition of Soybean Trypsin Inhibitor (Gibco Invitrogen Corporation) at a concentration of 10 mg/ml, cells were pelleted, washed and suspended in complete serum-free keratinocyte medium supplemented with 100 IU/ml penicillin, 100 μg/ml streptomycin, and 0.5 μg/ml Amphotericin B (Gibco Invitrogen Corporation). Approximately 3 × 10^6^ cells, with a viability above 95% using trypan blue exclusion, were seeded in 15 ml culture medium in each T-75 culture flask (Falcon®, Corning Life Sciences, Tweksburry, MA, USA) and cultured at 37°C, 6% CO_2_. The culture medium was replaced every second or third day until the cell confluence reached ~75%, within 19–28 days depending on skin donor. The cells were then detached with 0.05% Trypsin-EDTA treatment for 5 min at 37°C, followed by Soybean Trypsin Inhibitor 10 mg/ml, washed in culture medium, and thereafter ~5 × 10^6^ cells in 2 ml per vial were frozen using 10% DMSO and 50% heat inactivated fetal calf serum depleted from EV by ultracentrifugation and stored at −150°C.

### Co-cultures of *M. sympodialis* or MalaEx With Human Primary Keratinocytes

After thawing in a 37°C water bath for 1 min, the keratinocytes were transferred to a 50 ml tube and 18 ml of culture medium was slowly added. Thereafter, the keratinocytes were spun down, 180 × g for 7 min, and re-suspended in culture medium. Approximately 3 × 10^6^ cells, viability 80–90%, were seeded in 15 ml culture medium in each T-75 culture flask and cultured as above until the cell confluence, after 7–14 days, had reached ~75%. The cells were detached with 0.05% Trypsin-EDTA (see above), counted and seeded into μ-slides with 8 wells, 1 cm^2^ growth area per well, with a glass coverslip bottom (Cat. No. 80827, Ibidi, Martinsried, Germany). 0.6 × 10^5^ keratinocytes in 0.35 ml culture medium were seeded into each well. The μ-slides were incubated for 2–4 days at 37°C, 6% CO_2_ until 75% confluency was reached. Thereafter, the culture medium was removed and replaced with 0.35 ml fresh medium, and the stimulation agents were added to the keratinocytes; *M. sympodialis* (0.6 × 10^5^ and 3 × 10^5^ live yeast cells/well) harvested from the mDixon agar plates after 2 days of culture and the pooled MalaEx (1, 10, and 50 μg/ml). Lipopolysaccharides (LPS) (10 μg/ml, L8274, Sigma-Aldrich, Steinheim, Germany) was used as a positive control (Marcatili et al., 1997) and keratinocytes cultured in only the medium as a negative control. The cultures were incubated at 37°C, 6% CO_2_ for 24 h. The cells in the μ-slides were used for further analyses (see below).

### Confocal Laser-Scanning Microscopy (CLSM)

The keratinocytes co-cultured with *M. sympodialis*, MalaEx or LPS, or cultured alone in the 8 well μ-slides with a glass coverslip bottom were directly fixed in 4% formaldehyde for 10 min, treated with 0.5% Triton-X-100 (BDH Laboratory Supplies, Poole, UK) for 5 min, and blocked with 5% bovine serum albumin (BSA, Sigma-Aldrich) for 5 min at RT. Thereafter, Alexa Fluor 488 mouse anti-CD54/ICAM-1 or Alexa Fluor 488 IgG_1_ isotype control (Biolegend, San Diego, CA, USA), both at 1:20 dilution in 5% BSA, were added for 1 h at RT. The glass coverslip bottoms were finally covered with Prolong Gold antifade mountant (Invitrogen, Thermo Fisher Scientific, MA, USA). Fluorescent images as z-scans and phase contrast images were acquired on a CLSM (TCS SP2; Leica Microsystems, Mannheim, Germany). Each florescent image was combined with each corresponding phase contrast image. Confocal images were used to manually calculate the % of ICAM-1 positive keratinocytes defined as strongly positive. For each culture condition 100 cells were analyzed.

### Statistical Analysis

Statistical differences of stimulated keratinocytes compared to unstimulated was assessed by paired *t*-test using GraphPad Prism software (GraphPad Software, Inc. https://www.graphpad.com/scientific-software/prism/). Data are expressed as mean ± standard error of the mean (SEM). Differences were considered significant when *p* < 0.05.

## Results

### Characterization of MalaEx

**Figure 1:**
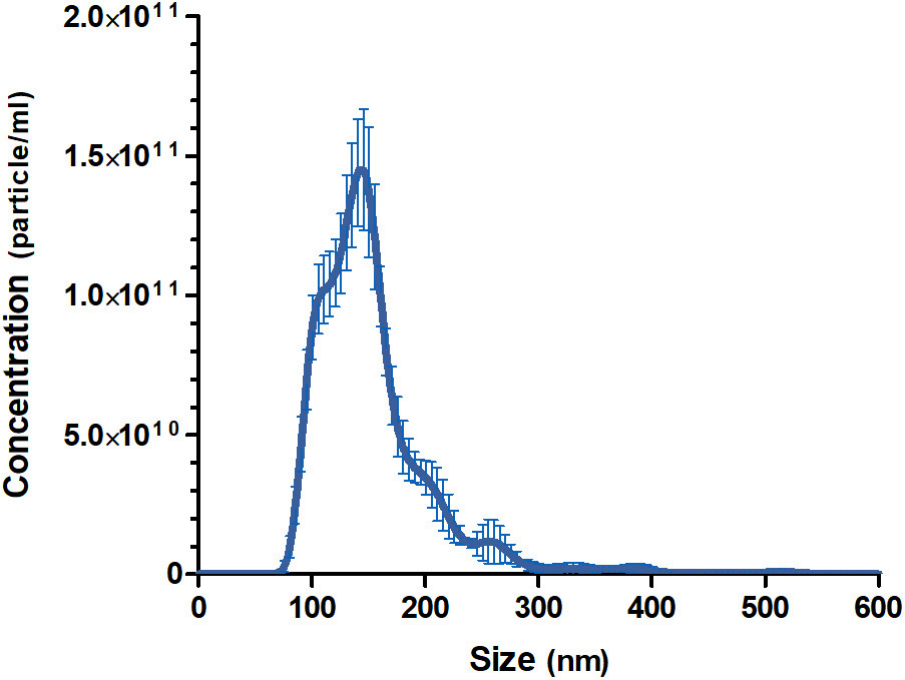
Size distributions and particle concentration of the pooled MalaEx preparation. Three MalaEx preparations were pooled and the pooled sample was investigated by nanoparticle tracking analysis (NTA) using NanoSight. The mean particle size and the particle concentration of four separate NTA runs of the pooled MalaEx sample are plotted. Error bars represent SD.

The pooled batch of three MalaEx batches harvested from 48 h *M. sympodialis* culture supernatants had a protein content of 1.45 mg/ml. NTA analysis indicated that the MalaEx had a size range of 70–580 nm, with a mean of 154 nm (*n* = 4 video recordings) and the total particle concentration was 1.19 × 10^13^ particles/ml (Figure 1).

### MalaEx Induce ICAM-1 Expression of Keratinocytes

**Figure 2:**
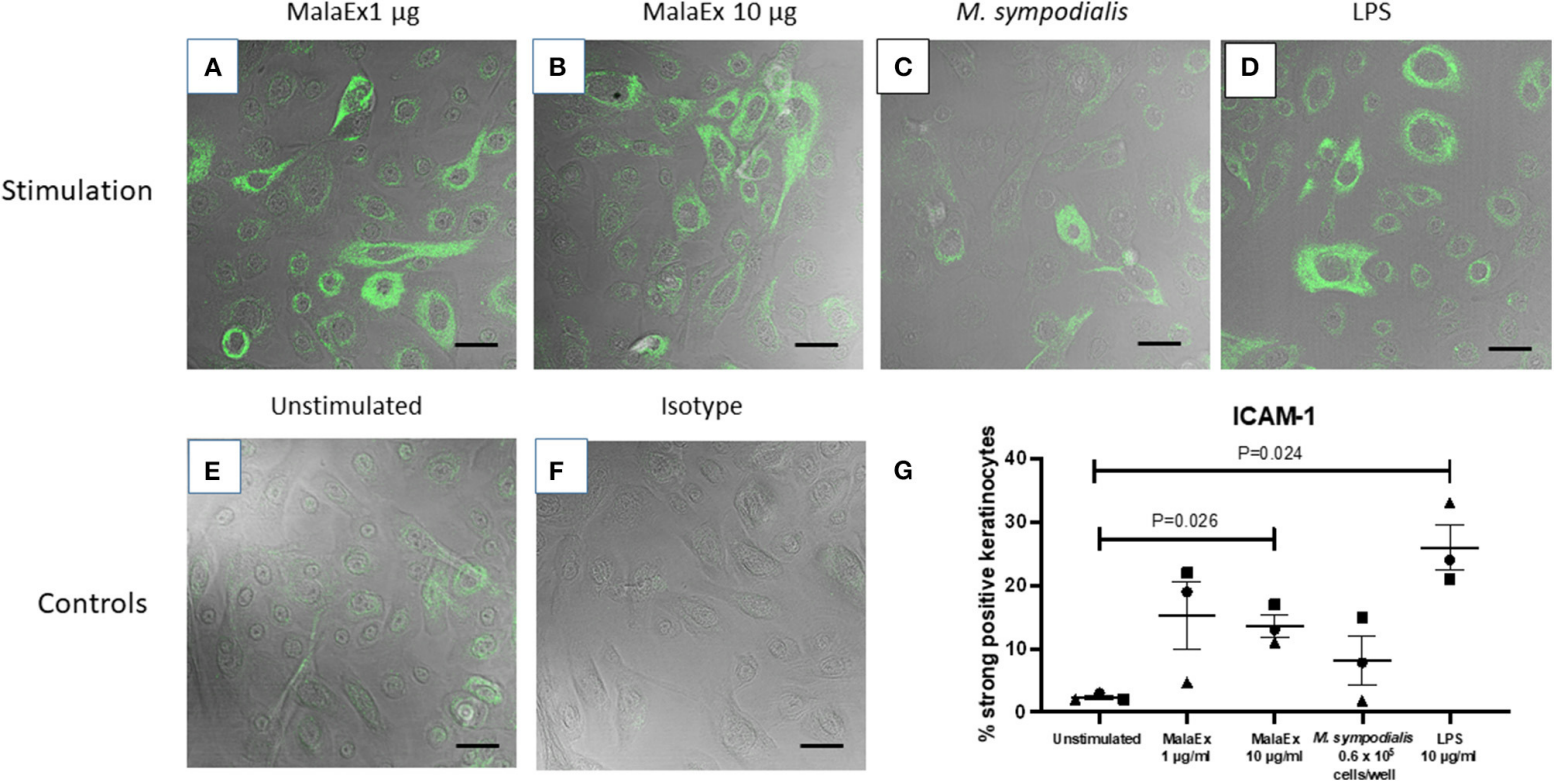
ICAM-1 expression of keratinocytes co-cultured with MalaEx. **(A–F)** Confocal microscopy with overlapping fluorescence and phase contrast images showing ICAM-1 expression using Alexa Fluor 488 mouse anti-CD54/ICAM-1 (green) of keratinocytes from donor 1 cultured for 24 h at 37°C with MalaEx at **(A)** 1 μg/ml, **(B)** 10 μg/ml, and **(C)** with *M. sympodialis* (0.6 × 10^5^ yeast cells/well), **(D)** with LPS (10 μg/ml) as a positive control, or **(E)** cultured alone. **(F)** Keratinocytes cultured alone where the isotype control Alexa Fluor 488 IgG_1_ has replaced the anti-ICAM-1 antibody. Size bars are 40 μm. **(G)** The % of ICAM-1 positive keratinocytes defined as strongly positive, after cultured alone or co-cultured with MalaEx (1 or 10 μg/ml) or *M. sympodialis* (0.6 × 10^5^ yeast cells/well) for 24 h at 37°C. LPS (10 μg/ml) was used as a positive control. Results are presented from three different keratinocyte donors (• Donor 1, ▴ Donor 2, and ■ Donor 3) as mean and standard error of the mean (SEM). Statistically significant differences, as calculated with paired *t*-test, are indicated in the figure.

Co-cultures of keratinocytes with MalaEx was performed for 24 h on μ-slides with 8 wells and a glass coverslip bottom enabling direct analysis with CLSM. Unstimulated keratinocytes showed that around 2% of the cells had a strong expression of ICAM-1 (Figure 2). MalaEx were found to induce an intense ICAM-1 expression on around 5–22% of the keratinocytes from the three different skin donors with a significant difference for the higher concentration of MalaEx, 10 μg/ml (mean ± SEM, 13.7 ± 1.8%, *p* = 0.026), as did the positive LPS control (mean 26 ± 3.6%, *p* = 0.024), compared to the unstimulated keratinocytes (Figure 2). We also tested addition of 50 μg/ml of MalaEx without any additional increase in ICAM-1 expression on the keratinocytes (data not shown). Co-culture with *M. sympodiali*s whole yeast cells (0.6 × 10^5^ cells/well) resulted in an upregulation of ICAM-1 expression to 8–15% of the keratinocytes from two of the donors; the donor who did not respond was the same who had a low response to 1 μg/ml of MalaEx (Figure 2). The 5-fold higher concentration, 3 × 10^5^ live yeast cells/well, gave similar results (data not shown).

## Discussion

The microbial flora on the skin constantly interact with the host skin barrier including immune competent cells in a complex manner influencing local and systemic immunity (Pasparakis et al., 2014). In the current study, we addressed whether nanovesicles, MalaEx, released by a commensal yeast on the skin, *M. sympodialis*, have capacity to modify keratinocytes. We found that MalaEx can activate keratinocytes to increase the expression of ICAM-1.

The MalaEx preparation harvested by a combination of centrifugation, filtration, and serial ultracentrifugation from supernatants from *M. sympodialis* had a size range of 70–580 nm in diameter, with a mean of 154 nm, similar to our previous reports on MalaEx (Rayner et al., 2017; Johansson et al., 2018). Our data are comparable with other studies on fungal EVs considering that different protocols for isolation of EVs and the various methods used for their analysis contribute to variations in reported sizes of fungal EVs (see Table 1 in Bielska and May, 2019). With cryo-electron tomography we have previously demonstrated that MalaEx have different sizes and are morphological diverse with varying electron-dense material suggesting different amount of internal content (Johansson et al., 2018). The heterogeneity of EVs most likely reflects distinct mechanisms of biogenesis, release, and functions (Soares et al., 2017). Our previous observation that MalaEx were mainly found in close proximity of the keratinocyte nuclei after internalization in keratinocytes, using super-resolution fluorescence 3D-SIM imaging, suggested a microbe-host communication (Johansson et al., 2018). We therefore in the present study investigated the expression of the activation marker ICAM-1 on the keratinocytes after co-cultures with MalaEx.

ICAM-1 is the best characterized inducible adhesion molecule on epithelial cells and is a counter ligand for the leukocyte integrins lymphocyte function-associated antigen-1 (LFA-1) and macrophage-1 antigen (Mac-1) (Smith et al., 1989). In the skin enhanced ICAM-1 expression on keratinocytes precedes dermal T lymphocytic infiltration (Griffiths and Nickoloff, 1989), and treatment with monoclonal antibodies to ICAM-1 or LFA-1 inhibits cell infiltration in contact sensitivity reactions in sensitized mice (Scheynius et al., 1993). We here found that only around 2% of the keratinocytes had a strong expression of ICAM-1 in the unstimulated cultures, in agreement with low constitutive expression of ICAM-1 on human keratinocytes (Dustin et al., 1988). MalaEx were capable to induce a 6-fold increase of strong ICAM-1 expression on primary human keratinocytes, and two of the keratinocyte donors responded also to *M. sympodiali*s whole yeast cells with increased ICAM-1 expression compared to the unstimulated keratinocytes (Figure 2). Inducible ICAM-1 expression on human keratinocytes is highly variable between different donors (Middleton and Norris, 1995), which can explain why one donor, nr 2, did not respond to the concentration *M. sympodiali*s whole yeast cells used, nor to the lower concentration of MalaEx (Figure 2). Further studies will explore the donor variation in response to MalaEx.

Increased ICAM-1 expression on human keratinocytes can be induced by LPS (Marcatili et al., 1997), IFN-ɤ (Dustin et al., 1988), TNF-α, and ultraviolet radiation (Krutmann et al., 1990). We could detect significantly increased ICAM-1 expression on the keratinocytes when the keratinocytes were co-cultured with MalaEx or LPS compared to the unstimulated keratinocytes (Figure 2). The kinetics and molecular mechanisms underlying the MalaEx induced upregulation of ICAM-1 expression on the keratinocytes are presently unclear and also whether other EVs (Bielska et al., 2019), have similar capacity. A recent review article highlights that our knowledge of the comparability of EVs in connecting different kingdoms is limited (Woith et al., 2019). Regarding capacity to participate in an allergic immune response we have previously, however, demonstrated that *M. sympodialis* released MalaEx can carry allergens similar to human dendritic or B-cell-derived exosomes and that all three types of EVs could induce Th2-like cytokine responses in blood cells from allergic donors (Admyre et al., 2007; Gehrmann et al., 2011; Vallhov et al., 2015).

A strength in the present study is the decision to mimic the situation on human skin as much as possible. We therefore used human primary keratinocytes from different donors, all in early passage. Cell lines, can behave considerable different compared with primary keratinocytes (Middleton and Norris, 1995), which is also the case for murine keratinocytes (Grone, 2002), and animal models with anatomical and immunological differences compared with humans (Di Meglio et al., 2011). At the same time our decision was a challenge, since the number of skin donors was limited, they were to us anonymous, and variations between human donors is expected (Middleton and Norris, 1995). For a more *in vivo* like situation, we also included the mixture of different MalaEx released into the supernatants of *M. sympodialis*, all assumed to be present on the skin. To obtain enough MalaEx we made a pool to avoid bias between different batches. Another strength is that the co-cultures of keratinocytes with MalaEx was performed on μ-slides with a glass coverslip bottom allowing direct analysis with CLSM, whereby *in vitro* manipulations to detach and separate the keratinocytes for further analysis were avoided. A limitation is, however, the use of mono-layer cultures of keratinocytes not reflecting the structure with distinct different layers of keratinocytes and appendages in normal skin. In future studies, human skin explants could be considered as a suitable model for superficial fungal infections (Corzo-Leon et al., 2019).

The ratio of *Malassezia* cells or their EVs to the number of keratinocytes in different *in vitro* studies are difficult to compare and relate to the complex *in vivo* situation in healthy vs. inflammatory skin conditions. To unravel the presence and biological significance of different EVs *in vivo* detailed studies are needed requiring development of new technologies (Coelho and Casadevall, 2019; Margolis and Sadovsky, 2019). In agreement with our observation that keratinocytes can be activated by fungal EVs *in vitro* is a study where EVs from the dermatophyte *Trichophyton interdigitale* induced the release of proinflammatory mediators by the human keratinocyte line HaCat after 24 co-culture (Bitencourt et al., 2018). Recently, EVs from *Malassezia furfur* were found capable to stimulate IL-6 production in HaCaT cells and mice epidermal keratinocytes (Zhang et al., 2019), strongly supporting our results that *Malassezia* EVs, not only their parental whole yeast cells (Watanabe et al., 2001; Ishibashi et al., 2006; Donnarumma et al., 2014), have the capacity to activate keratinocytes. Furthermore, they presented promising *in vivo* evidence in a mouse model showing that EVs from *M. furfu*r topically applied could penetrate the skin and induce IL-6 expression on the keratinocytes as analyzed with immunohistochemical staining on skin sections (Zhang et al., 2019). Notably, they also confirmed our observation (Johansson et al., 2018) on internalization of EVs by keratinocytes and their perinuclear distribution pattern (Zhang et al., 2019).

Potential beneficial effects to harbor the commensal yeast *Malassezia* on the skin should not be neglected to elucidate. In that line, a unique secreted as partyl protease produced by *Malassezia globosa*, MgSAP1, was discovered to hinder *Staphylococcus aureus* biofilm formation, an established virulence attribute of *S. aureus* (Li et al., 2018). This study invites for investigations if fungal EVs have a role in release and transport of such molecules in microbial interactions and their possible beneficial effects on the host (Ianiri et al., 2018).

## Conclusions

In conclusion, our data show that *M. sympodialis* released nanosized MalaEx are able to activate human keratinocytes with an enhanced ICAM-1 expression. This implies a possible cross-kingdom modulation of host cells where activation of keratinocytes by MalaEx may be an important first step in cutaneous defense to *M. sympodialis*. How this modulation of host cells by a fungus is balanced between the commensal, pathogenic or beneficial states on the skin in the interplay with the host needs to be further elucidated.

## Data Availability Statement

The raw data supporting the conclusions of this article will be made available by the authors, without undue reservation, to any qualified researcher.

## Ethics Statement

The study was approved by the regional ethical review board in Stockholm (2015/2082-31/1). Written informed consent was obtained at Strandkliniken, a local plastic surgery clinic in Stockholm, from all subjects donating skin. The donors were anonymous to the recipients of the skin. All experiments were performed in accordance with the Helsinki Declaration ethical principles for medical research.

## Author Contributions

HV, CJ, and AS conceptualized and designed the study. HV and CJ performed the co-culture experiments. RV did the NTA analysis. CJ worked on the statistical analysis. AS wrote the manuscript together with HV. CJ and RV wrote sections of the manuscript. All authors contributed to manuscript revision, read, and approved the submitted version.

### Conflict of Interest

AS is a member in the Joint Steering Committee for the Human Translational Microbiome Program at SciLifeLab/Karolinska Institutet together with Ferring Pharmaceuticals, Switzerland. The remaining authors declare that the research was conducted in the absence of any commercial or financial relationships that could be construed as a potential conflict of interest.
